# Integrated miRNA Sequencing and Network Analysis Reveal a Molecular Continuum Between Peritumoral and Tumor Tissue in Prostate Cancer

**DOI:** 10.3390/ijms27156637

**Published:** 2026-07-25

**Authors:** Rafael Parra-Medina, Elizabeth Vargas-Castellanos, Dayana Rodríguez-Morales, Sandra Ramírez-Clavijo, Jovanny Zabaleta, César Payán-Gómez

**Affiliations:** 1 Pathology Department, Instituto Nacional de Cancerología (INC), Bogota 111511, Colombia; 2 Research Institute, Universidad FUCS, Bogotá 111221, Colombia; 3 Hospital Universitario Mayor—Méderi, Universidad del Rosario, Bogota 110321, Colombia; 4 Department of Biology, Faculty of Natural Sciences, Universidad del Rosario, Bogota 111221, Colombia; 5 Department of Interdisciplinary Oncology, Louisiana State University Health Science Center (LSUHSC), New Orleans, LA 70112, USA; 6 Dirección Académica, Universidad Nacional de Colombia, Sede de La Paz, La Paz 201501, Colombia

**Keywords:** cancer, prostate, peritumoral tissue, miRNA

## Abstract

Field cancerization describes molecular alterations occurring in histologically normal tissues surrounding tumors that may contribute to cancer initiation and progression. In prostate cancer (PCa), the molecular characteristics of peritumoral tissue (PTT) remain incompletely understood. Because microRNAs (miRNAs) play key roles in gene regulation, tumor progression, and microenvironmental remodeling, we investigated miRNA expression patterns and regulatory networks across benign tissue (BT), PTT, and tumor tissue (TT). Small RNA sequencing was performed on matched formalin-fixed paraffin-embedded samples from 40 patients with PCa. Differential expression analysis was conducted using DESeq2, adjusting for age and Gleason grade, while functional enrichment analysis and weighted gene co-expression network analysis (WGCNA) were used to identify dysregulated pathways and conserved miRNA modules. PTT exhibited a molecular profile intermediate between BT and TT, consistent with a field cancerization effect. Compared with BT, 102 miRNAs were differentially expressed in TT and 57 in PTT, with 39 miRNAs (68% of the PTT-associated miRNAs) overlapping the tumor signature. Shared dysregulated pathways included PI3K–Akt, p53, and HIF-1 signaling; whereas, PTT showed additional enrichment in pathways related to epigenetic regulation (Polycomb Repressive Complex) and cellular stress responses (mitophagy, protein processing in ER) exclusively through up-regulated miRNAs; no pathways were uniquely enriched from down-regulated miRNAs in PTT. WGCNA identified conserved miRNA modules enriched for members of the let-7, miR-200, miR-103/107, and miR-106a~363 families, which have established roles in epithelial–mesenchymal transition, tumor progression, and microenvironmental remodeling. Collectively, these findings demonstrate that histologically benign peritumoral tissues harbor tumor-associated miRNA programs and regulatory networks that closely resemble those observed in prostate tumors, providing molecular evidence of field cancerization in PCa and identifying potential miRNA-mediated mechanisms relevant to disease progression and biomarker development.

## 1. Introduction

Prostate cancer (PCa) is a major public health concern worldwide, with more than 1.5 million newly diagnosed cases and 397,430 deaths in 2022 [1]. PCa is a tumor with a high degree of heterogeneity in the composition of the tumor microenvironment (TME), including cancer-associated fibroblasts, stromal cells, extracellular matrix, and immune cells. In contrast to other tumors, the TME of PCa has been described as an immunosuppressive state which may be due to poor cytolytic activity of NK cells, increased TGF-β secretion by prostate tissue, and reduced antitumor immunity from activated T regulatory cells [2,3].

The peritumoral tissue (PTT), also referred to as normal tissue adjacent to the tumor (NAT), represents the interface between the tumor and the surrounding histologically normal tissue [4]. Historically, PTT has been considered normal tissue and has frequently been used as a control for tumor tissue under the assumption that histological normality reflects biological normality. However, previous studies have demonstrated that PTT can harbor molecular alterations at the genetic, epigenetic, transcriptional, and post transcriptional levels, as well as dysregulated cellular processes including proliferation, metabolism, inflammation, DNA repair, and stromal–epithelial interactions [5]. These molecular changes occurring in histologically normal tissues surrounding the tumor are referred to as “field cancerization” or the “field effect” [6,7].

Histologically PTT exhibits molecular alterations despite the absence of morphological abnormalities, supporting the concept of field cancerization. Aran et al. [8]. performed a comprehensive transcriptomic analysis of 6506 samples from the Genotype-Tissue Expression (GTEx) project and The Cancer Genome Atlas (TCGA), encompassing eight tumor types (lung, colon, breast, uterus, liver, bladder, prostate, and thyroid). Their study demonstrated that PTT represents a distinct intermediate molecular state between normal and tumor tissue, characterized by widespread differential gene expression, activation of inflammatory and stress-response pathways, and extracellular matrix remodeling. Furthermore, they proposed that tumor-derived pro-inflammatory signals may induce transcriptional changes in the adjacent endothelium, highlighting an active molecular interaction between the tumor and its surrounding tissue. PTT interacts with tumor cells and can control the tumor microenvironment with an impact on the progression, immune evasion, and treatment response [9]. This control involves the remodeling of the extracellular matrix, the presence of fibrosis, the modification of the wound response pathway, and increase in the local epithelial–mesenchymal transition (EMT) [10]. It has also been shown in recent years that PTT microenvironment modulates pro- and anti-tumor immunity, with differential infiltration of fibroblast, epithelial cells, cancer-associated fibroblast, T cells (CD8+, Th1, Treg), macrophages (M2), neutrophils, and NK cells [9].

microRNAs (miRNAs) are small (~20–22 nucleotides) non-coding RNAs that post-transcriptionally regulate gene expression through mRNA degradation or translational repression, thereby controlling multiple biological processes involved in tissue homeostasis and tumorigenesis [11,12]. Increasing evidence indicates that miRNAs are embedded within complex post-transcriptional regulatory networks involving long non-coding RNAs (lncRNAs), which contribute to the regulation of gene expression and multiple cancer-related signaling pathways [13,14]. In PCa several miRNAs have been described in the pathogenesis of the disease, having the potential of serving as biomarkers in diagnosis, treatment and prognosis [15,16].

In recent years, PTT has been associated with early tumorigenesis, metastatic potential, recurrence, treatment response, and overall prognosis [17,18,19]. Moreover, the expression of miRNAs in PTT has also been linked to patient prognosis in malignant tumors [20]. Therefore, in this study, we employed next-generation sequencing alongside weighted gene co-expression network analysis (WGCNA) to comprehensively characterize miRNA expression profiles across benign tissue (BT), tumoral tissue (TT), and peritumoral tissue (PTT) for formalin-fixed, paraffin-embedded (FFPE) tissue from patients with prostate cancer. The aim was to elucidate the molecular continuity among these compartments and to identify both shared and state-specific transcriptional programs.

## 2. Results

### 2.1. Patient Cohort Characteristics

A total of 40 patients with histologically confirmed prostate adenocarcinoma were initially included, and all available tumor, adjacent, and benign tissue samples underwent RNA sequencing (RNA-seq). After quality control, three patients were excluded, resulting in a final cohort of 37 patients with complete paired tumor, adjacent, and benign tissue samples. Clinicopathological characteristics for these 37 patients are summarized in Table 1. The median age at diagnosis was 65.4 years (range 48.2–79.1), and the median PSA level was 10.3 ng/mL (range 2.4–43.0). Gleason grade groups were distributed as follows: group 1, 6 (16.2%); group 2, 8 (21.6%); group 3, 7 (18.9%); group 4, 8 (21.6%); and group 5, 8 (21.6%). Perineural invasion was present in 22 patients (59.5%), and extraprostatic extension in 23 (62.2%). At the time of analysis, 28 patients (75.7%) were alive, five (13.5%) were deceased, and vital status was unavailable for four (10.8%).

### 2.2. Global miRNA Expression Reveals a Transcriptional Continuum from Benign to Tumor via Peritumoral Tissue

**Figure 1:**
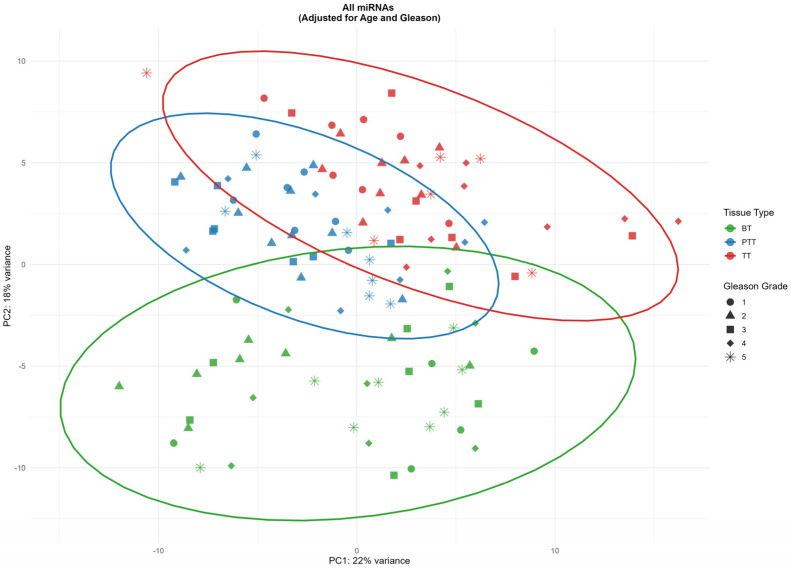
Global miRNA expression profiling distinguishes TT, PTT and BT. PCA of miRNA expression profiles. PC1 and PC2 explain 22% and 18% of variance, respectively. PC2 separates benign samples (negative side) from tumor and peritumoral samples (positive side). PC1 partially separates the tumor (positive side) from peritumoral (negative side). Peritumoral samples are positioned between benign and tumor.

Principal Component Analysis (PCA) on normalized miRNA counts (37 TT, 39 PTT, 37 BT) is shown in Figure 1. The first two principal components explained 40% of the total variance (PC1 22%, PC2 18%). PC2 separated TT and PTT samples from BT: most TT and PTT samples clustered on the positive side, while BT samples clustered on the negative side. PC1 partially separated tumor from PTT: most TT samples clustered on the positive side, and most PTT samples on the negative side. Together, the two components provided a partial separation of the three tissue types, with PTT samples positioned between benign and tumor.

**Figure 2:**
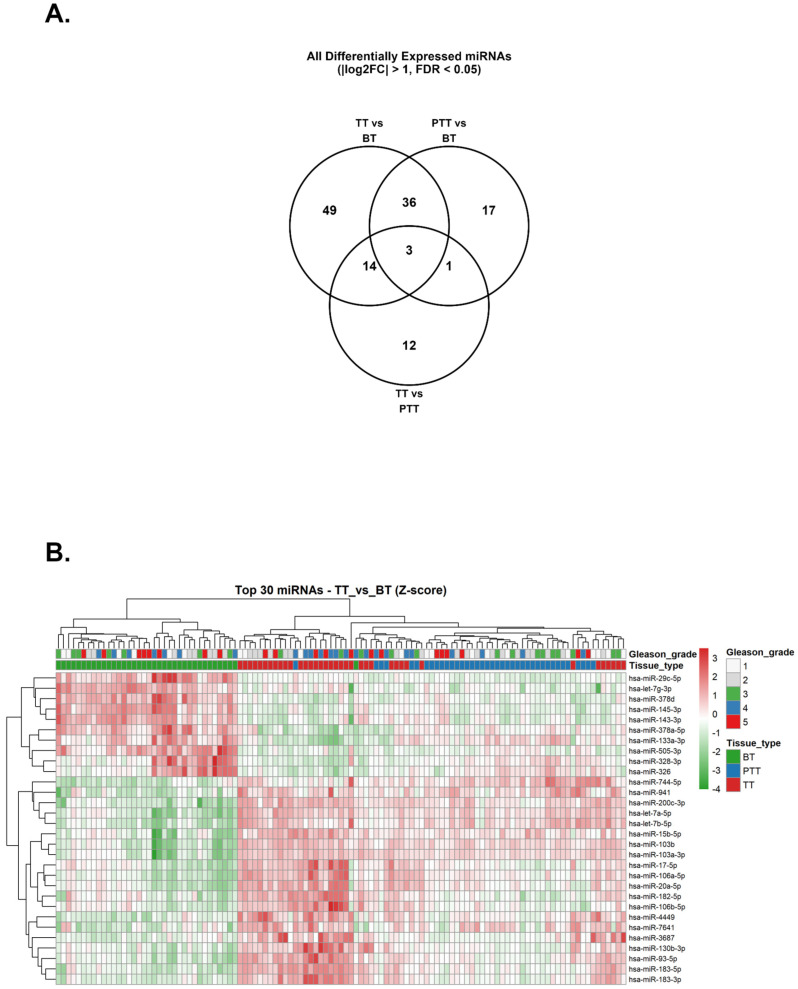
Differential miRNA expression reveals shared and tumor-specific alterations. (**A**) Venn diagram showing the number of DEmiRNAs identified in each pairwise comparison: (i) TT vs. BT; (ii) PTT vs. BT; (iii) TT vs. PTT. A total of 39 miRNAs are common to both TT vs. BT and PTT vs. BT comparisons, representing 68% of the PTT signature. All shared miRNAs exhibit concordant regulation. (**B**) Two-way hierarchical clustering of the 30 most significant DEmiRNAs from the TT vs. BT comparison. Rows represent miRNAs (Z-score normalized expression), columns represent individual samples (TT, PTT, BT). The horizontal dendrogram shows sample clustering; the vertical dendrogram separates down-regulated (upper cluster) and up-regulated (lower cluster) miRNAs.

Differential expression analysis (|log_2_FC| ≥ 1, FDR < 0.05) was performed for three pairwise comparisons: (i) TT vs. BT; (ii) PTT vs. BT; (iii) TT vs. PTT (Figure 2A). Tumor tissue exhibited extensive miRNA dysregulation (102 miRNAs), while PTT exhibited 57 miRNAs when compared to BT, with a substantial overlap (68%) with TT, supporting a shared regulatory landscape. Of these, 39 miRNAs (68% of the PTT differential expression) were common to both comparisons and showed concordant regulation (up-regulated in both or down-regulated in both). Forty-nine miRNAs (57% of the tumor signature) were dysregulated exclusively in TT. Direct comparison between TT and PTT revealed 30 differentially expressed miRNAs, of which 12 were also significantly altered in TT vs. BT and PTT vs. BT comparisons, while one was unique to the TT vs. PTT comparison.

Hierarchical clustering of the 30 most significant miRNAs from the TT vs. BT comparison (Figure 2B) placed PTT samples in an intermediate position. The sample dendrogram separated BT samples from a large branch containing all tumor and PTT samples. Within that branch, a pure TT subcluster, mixed subclusters, and a pure PTT subcluster were observed. Expression intensity (Z-score) showed a gradient: strongest in the pure TT subcluster, progressively milder in mixed and pure PTT subclusters.

### 2.3. Functional Overlap and Asymmetry in miRNA-Driven Pathways

**Figure 3:**
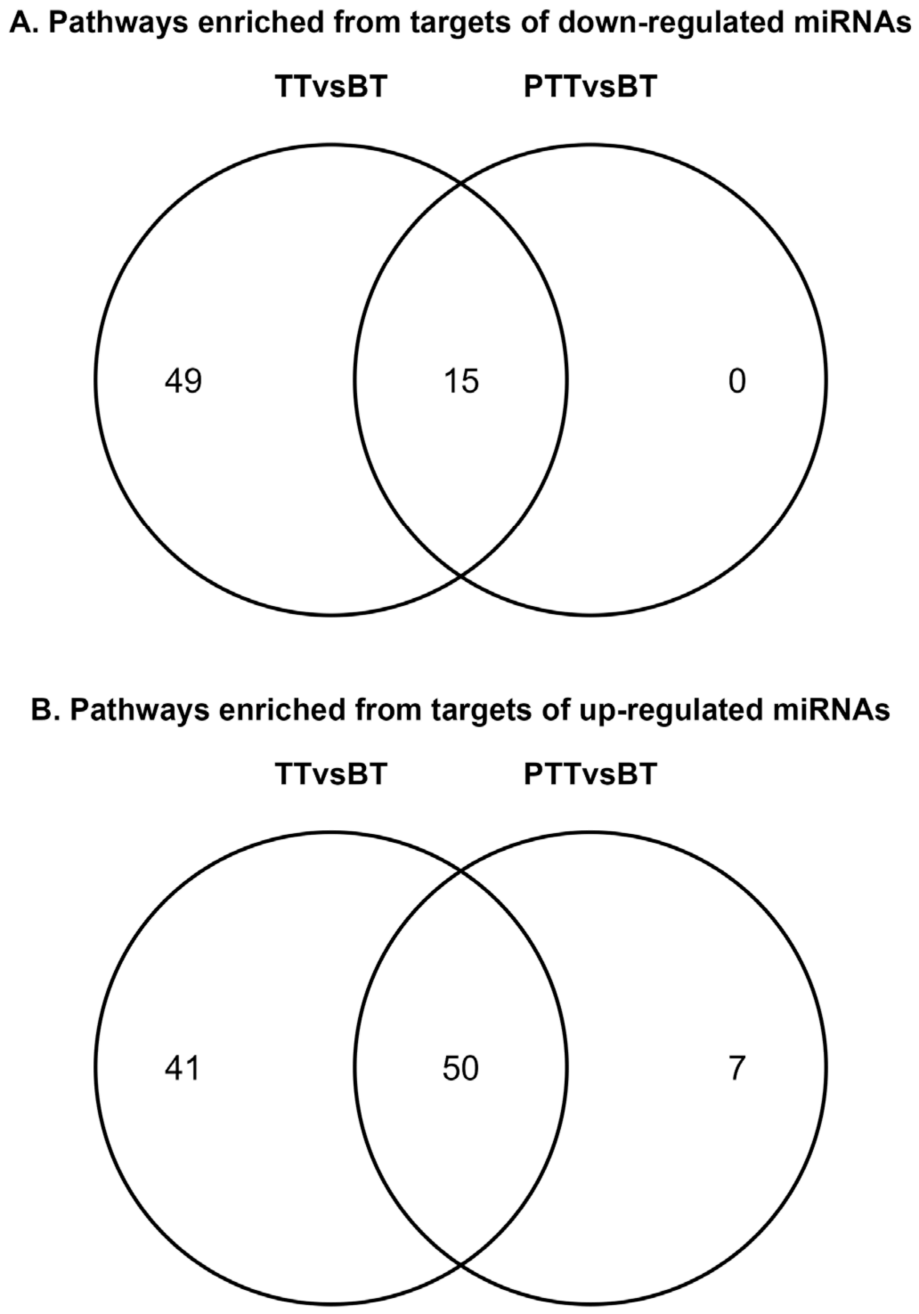
Comparative pathway enrichment analysis of miRNA targets in TT and PTT. Venn diagrams showing the number of significantly enriched KEGG pathways (FDR < 0.05) identified from the target genes of differentially expressed miRNAs. (**A**) Pathways enriched from targets of down-regulated miRNAs. (**B**) Pathways enriched from targets of up-regulated miRNAs. Full lists of pathways are provided in Tables S3 and S4.

Over-representation analysis (KEGG) was performed on experimentally validated target genes of differentially expressed miRNAs (targets from intersection of TargetScan, miRDB, miRTarBase). For down-regulated miRNAs in TT vs. BT, 49 KEGG pathways were enriched exclusively in this comparison (Table S1); 15 pathways were shared with PTT vs. BT, representing all pathways detected in PTT via down-regulated miRNAs (Figure 3A). No pathways were uniquely enriched from down-regulated miRNAs in PTT vs. BT.

For up-regulated miRNAs, 41 pathways were enriched exclusively in TT vs. BT (Table S2). In PTT vs. BT, 57 pathways were enriched, of which 50 were shared with TT vs. BT; the remaining seven were unique to PTT (Figure 3B). These seven pathways were: “Longevity regulating pathway—multiple species”, “Tight junction”, “Polycomb Repressive Complex”, “Mitophagy–animal”, “Protein processing in endoplasmic reticulum”, “Chemical carcinogenesis—receptor activation”, and “Transcriptional misregulation in cancer” (Table 2).

List of KEGG pathways significantly overrepresented (FDR < 0.05) among the target genes of miRNAs up-regulated exclusively in the PTT versus BT comparison. Target genes were obtained from the intersection of miRWalk predictions using TargetScan, miRDB, and miRTarBase. For each pathway, the number of target genes identified (Count), fold enrichment, and False Discovery Rate (FDR) are shown.

### 2.4. Weighted Gene Co-Expression Network Analysis Reveals Conserved and Specific miRNA Modules in TT and PTT

To more precisely characterize the transcriptional programs of miRNAs across the different tissue compartments we performed a WGCNA. This method complements conventional differential expression analysis by grouping miRNAs with highly correlated expression patterns into modules that often reflect coordinated biological pathways. By constructing separate networks for TT vs. BT and PTT vs. BT samples, we aimed to identify both shared and compartment-specific miRNA programs, thereby gaining a more integrated view of the molecular landscape underlying prostate cancer progression.

For WGCNA, miRNAs with |log_2_FC| ≥ 0.584 and FDR < 0.05 in at least one of the three differential comparisons were selected, yielding 226 miRNAs (Table S3). Two separate co-expression networks were built—TT vs. BT and PTT vs. BT—using soft-thresholding powers of β = 14 and β = 16, respectively (R^2^ > 0.8 for scale-free topology). Robust modules were identified in each network, and their eigengenes were correlated with tissue type.

**Figure 4:**
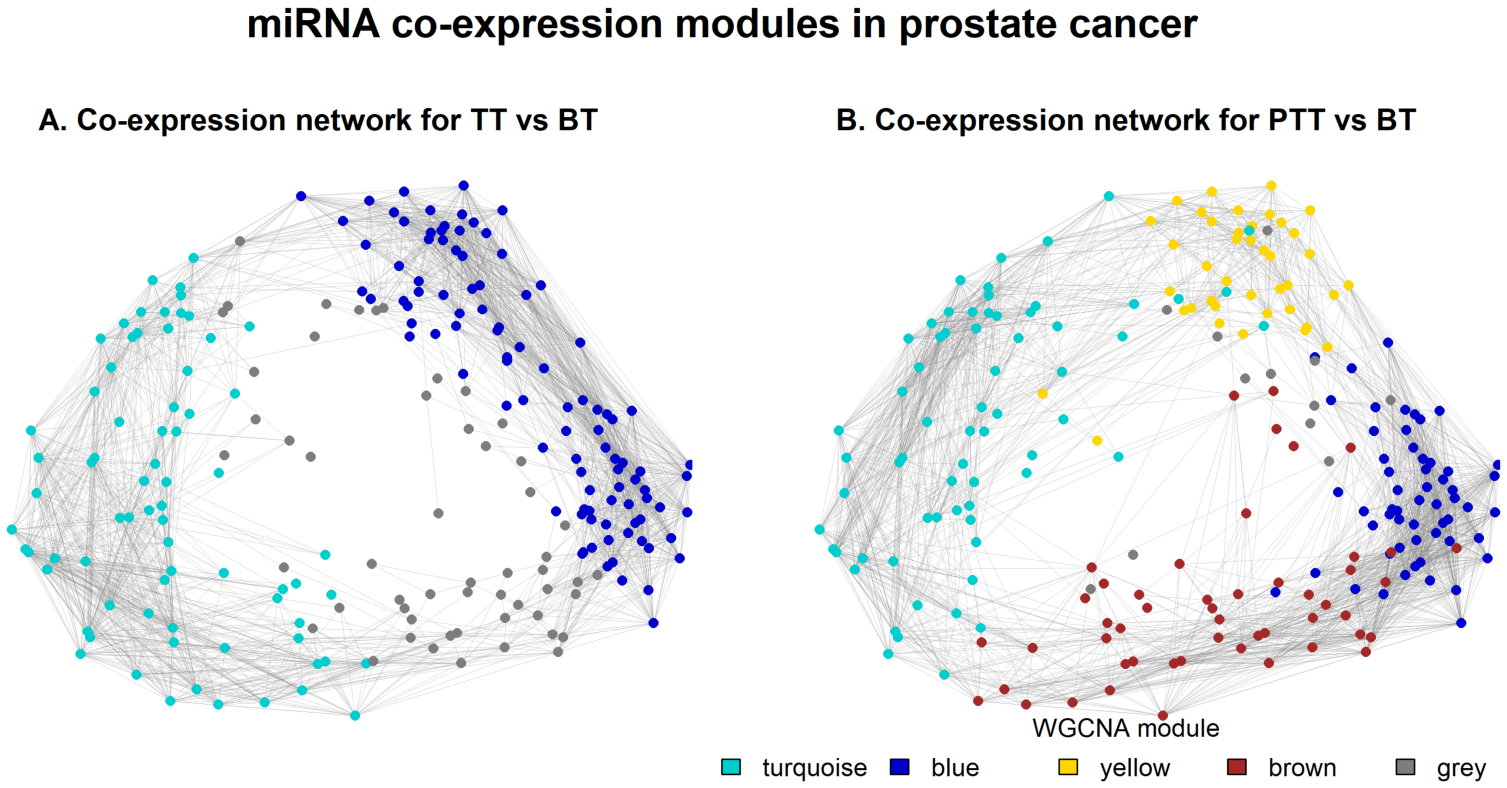
WGCNA co-expression networks for TT vs. BT and PTT vs. BT comparisons. Each network includes all miRNAs analyzed (226 for TT vs. BT; 224 for PTT vs. BT). Nodes represent miRNAs colored by WGCNA module (turquoise, blue, yellow, brown, gray). Edges show the strongest weighted adjacencies (top 10th percentile of (0.5 × (1 + cor))^power^); line transparency reflects edge strength. Node layouts were calculated with a force-directed algorithm using edge weights as attractive forces and increased repulsion to separate poorly connected nodes. In both networks, same-colored miRNAs form dense clusters, while unassigned gray nodes appear as a dispersed cloud.

In the TT network, two modules were identified: a turquoise module (73 miRNAs, positively correlated with tumor, r = 0.90, *p* < 0.001) and a blue module (99 miRNAs, negatively correlated, r = –0.85, *p* < 0.001). In the PTT network, four modules were detected: turquoise (66 miRNAs, r = 0.83, *p* < 0.001), blue (56 miRNAs, r = –0.85, *p* < 0.001), brown (50 miRNAs, r = –0.22, *p* > 0.05), and yellow (40 miRNAs, r = –0.21, *p* > 0.05) (Figure 4).

Module preservation and overlap statistics are summarized in Table 3. The turquoise modules of the two networks overlapped significantly: 52 of 66 PTT turquoise miRNAs (78.8%) were also present in the TT turquoise module (odds ratio 24.0, FDR 1.71 × 10^−20^). Similarly, the PTT blue module shared 49 of its 56 miRNAs (87.5%) with the TT blue module (odds ratio 16.6, FDR 3.03 × 10^−14^). Module preservation analysis (Zsummary) further indicated that these modules were moderately conserved (Zsummary = 9.96 and 6.39, respectively), suggesting that while their miRNA composition is maintained, their internal connectivity patterns undergo moderate rewiring in the tumor context. The PTT yellow module overlapped with the TT blue module (36 of 40 miRNAs, 90%; odds ratio 17.4, FDR 7.09 × 10^−11^); however, its poor preservation (Zsummary = 0.64) reveals that this peritumoral module is not structurally conserved in the tumor microenvironment despite the high overlap. The PTT brown module showed no significant overlap with any tumor module (FDR > 0.05).

### 2.5. Key Drivers of the Conserved Turquoise Module

**Figure 5:**
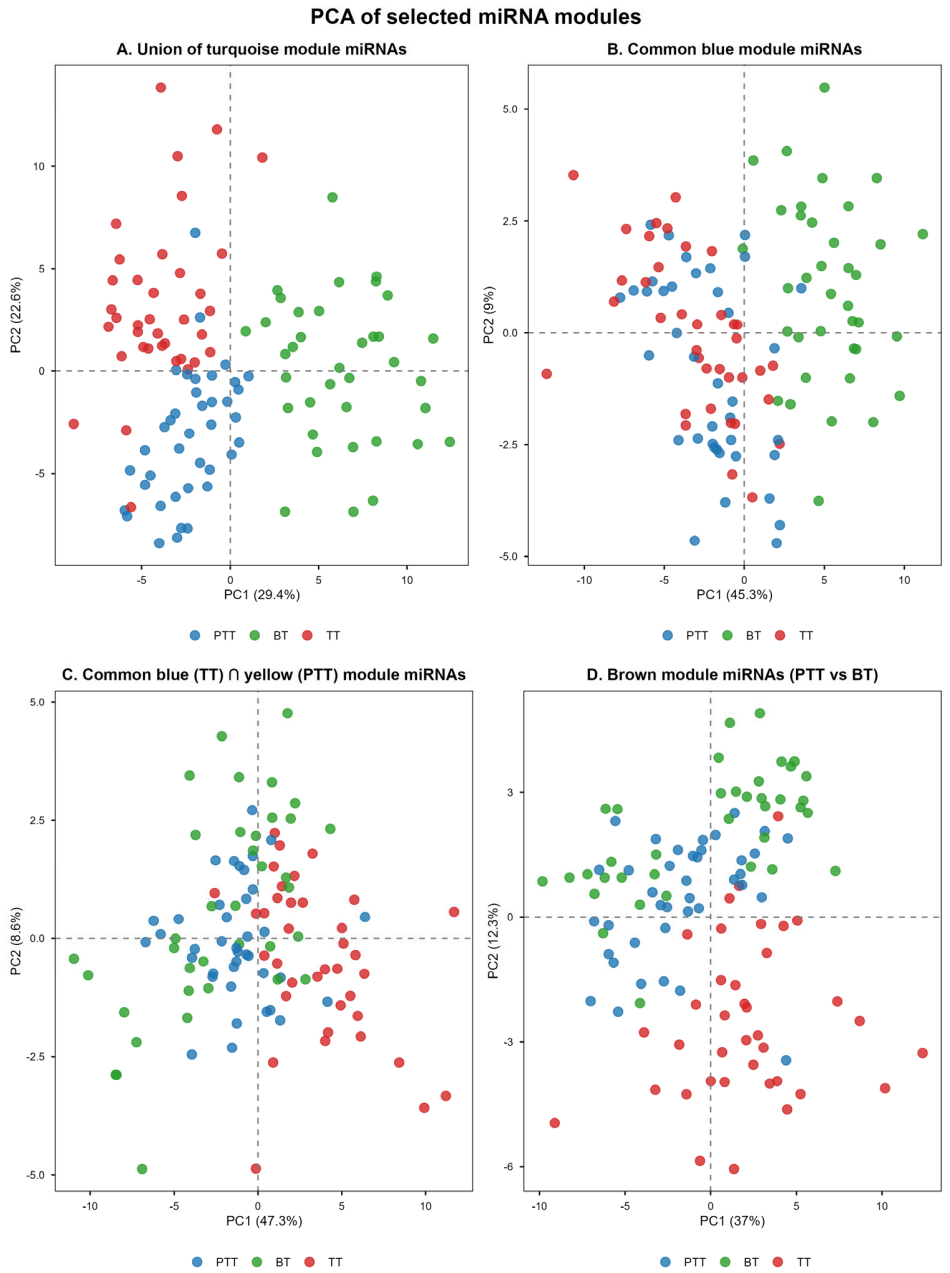
Principal Component Analysis of module-specific miRNAs reveals distinct transcriptional programs in TT, PTT and BTs. (**A**) Turquoise module PCA: Projection of samples onto PC1 and PC2 based on the normalized expression of 87 miRNAs from the conserved turquoise module. PC1 (29.4% of variance) separates BT samples (positive side) from TT and PTT (negative side). (**B**) Blue module PCA: Projection of samples onto PC1 and PC2 based on the normalized expression of 115 miRNAs from the blue module (TT network) and blue/yellow modules (PTT network). PC1 (45.3% of variance) separates benign samples (positive side) from TT and PTT samples (negative side). (**C**) Blue-yellow module intersection PCA: PCA of the 36 miRNAs shared between the tumor blue module (99 miRNAs) and the PTT yellow module (40 miRNAs). PC1 (45.3% of variance) separates TT samples (positive side) from PTT samples (negative side). BT samples are distributed across the axis, with a tendency toward the negative side. (**D**) Brown module PCA: Projection of samples onto PC1 and PC2 based on the normalized expression of 50 miRNAs from the PTT-specific brown module. PC2 separates miRNAs into two groups based on their loadings.

From the union of turquoise module miRNAs (87 miRNAs), PCA was performed (Figure 5A). PC1 (29.4% of variance) separated benign from TT/PTT samples. The nine miRNAs with the highest absolute loadings on PC1 (top 10%) were identified (Table S4). They belonged to the let-7 family, the miR-200 family together with the miR-103/107 cluster, and the miR-106a~363 cluster. All nine miRNAs were up-regulated in both TT and PTT relative to BT (negative loadings). Their target genes were enriched in 33 KEGG pathways (FDR < 0.05), including pathways in cancer, p53, FoxO, PI3K-Akt, HIF-1, and prostate cancer, among others (Table S5).

The second principal component of the same PCA (PC2) distinguished TT from PTT: TT samples predominantly on the positive side, PTT on the negative side (Figure 5A). The nine miRNAs with highest absolute loadings on PC2 were divided into four with negative loadings (higher in PTT) and six with positive loadings (higher in tumor) (Table S6). The six TT-up-regulated miRNAs targeted 14 KEGG pathways (FDR < 0.05), mainly involved in oncogenic signaling, cancer hallmarks, and drug resistance (Table S7). The four PTT-up-regulated miRNAs targeted seven pathways that showed nominal enrichment (*p* < 0.05) but did not survive FDR correction (Table S8).

### 2.6. A Tumor-Specific Repression Signature Distinguishes TT from PTT

The blue module was highly conserved between networks. In the PTT network, this program split into two modules: blue (56 miRNAs, r = –0.85) and yellow (40 miRNAs, r = –0.21). The yellow module overlapped extensively with the TT-blue module (90% shared). From the union of miRNAs in the TT-blue, PTT -blue, and PTT-yellow modules (115 miRNAs), PCA was performed (Figure 5B). PC1 (45.3% of variance) separated BT from TT/PTT samples. Five miRNAs with the highest loadings (top 10%) were identified (Table S9); all were down-regulated in both TT and PTTs (positive loadings). Their target genes were enriched for the FoxO signaling pathway (FDR < 0.05) and showed nominal enrichment for several other pathways (Table S10).

From the overlap of the TT-blue (99 miRNAs) and PTT-yellow (40 miRNAs) modules, 36 common miRNAs were retained. PCA on these 36 miRNAs (Figure 5C) showed that PC1 (45.3% of variance) separated TT samples (positive side) from PTT samples (negative side); BT samples were distributed along the axis, tending toward the negative side. The four miRNAs with the highest absolute loadings (all negative) were miR-328-3p, miR-887-3p, miR-326, and miR-133a-3p (Table S11). Negative loadings indicated higher expression in PAT and BT and lower expression in TT samples. Target genes of these four miRNAs were enriched in KEGG pathways including prostate cancer, Ras signaling, and endocrine resistance (FDR < 0.05), with additional pathways showing nominal enrichment (Table S12).

### 2.7. A PTT-Specific Module Captures a Dual Program in the PTT Microenvironment

The brown module (50 miRNAs) was unique to the PTT network and showed no significant overlap with any tumor module. PCA of these miRNAs (Figure 5D) revealed two distinct expression patterns along PC2. The six miRNAs with the highest absolute loadings on PC2 were divided into two groups: two with negative loadings (miR-182-5p, miR-25-3p) and four with positive loadings (miR-224-5p, miR-338-3p, miR-1-3p, miR-205-3p) (Table S13).

For the four positive-loading miRNAs, target genes (*n* = 10) were enriched in 24 GO terms (FDR < 0.05), covering angiogenesis, cell migration, axon guidance, growth factor signaling, and hormonal processes (Table S14). For the two negative-loading miRNAs, target genes (*n* = 33) were enriched in seven GO terms related to apoptosis regulation, cell growth, and sister chromatid cohesion (Table S15). KEGG analysis of the positive-loading miRNAs showed enrichment of the Axon guidance pathway (hsa04360) with nominal significance (FDR = 0.054) (Table S16).

## 3. Discussion

The present study demonstrates that PTT exhibits a molecular profile more similar to TT than to BT. Notably, several miRNAs and associated oncogenic pathways were shared between PTT and TT when compared to BT, suggesting that, despite its morphologically benign appearance, PTT harbors tumor-like molecular alterations. Principal Component Analysis and hierarchical clustering further positioned PTT in an intermediate state between BT and TT, supporting the concept of field cancerization at the miRNA level in PCa [7]. Differential expression analysis revealed that approximately 68% of the miRNAs altered in PTT were also dysregulated in TT, quantitatively highlighting the substantial molecular overlap between these tissues. This pattern, which has also been reported in other tumor types [21], reinforces the notion that PTT regions may represent a pre-neoplastic field characterized by early molecular changes that precede tumor development or contribute to disease recurrence.

Among the seven pathways uniquely enriched from the target genes of up-regulated miRNAs in PTT (Table 2), we found that some are related to epigenetic dysregulation driven by the Polycomb Repressive Complex (PRCs) and metabolic reprogramming, including changes in longevity-related pathways, mitophagy, and endoplasmic reticulum stress responses. These findings suggest that these pathways may be down-regulated in PTT because of miRNA-mediated repression, supporting the notion that the ‘field of cancerization’ is orchestrated by epigenetic changes that precede malignant transformation. PRC1 and PRC2 mediate gene repression through histone modifications such as H2AK119ub1 and H3K27me3, respectively, thereby maintaining transcriptional memory [22]. Dysregulation of this system, especially through overexpression of EZH2, the catalytic subunit of PRC2 has been associated with higher tumor grade and aggressive behavior in PCa, supporting its oncogenic role and suggesting that epigenetic repression may contribute to the transcriptional landscape observed in advanced disease [23].

The longevity pathway integrates key processes such as nutrient sensing, stress resistance, and cell survival, largely mediated by signaling axes like mTOR and insulin/IGF-1. These pathways are critical regulators of cellular energetics, proteostasis, and senescence, and their dysregulation may support tumor cell adaptation and persistence [24]. IGF-1 may contributes to PCa proliferation, survival, and therapy resistance by promoting tumor growth and migration through activation of integrin–FAK and Akt/mTOR signaling pathways in both androgen-dependent and androgen-independent cells [25]. Additionally, the involvement of tight junction components points to a disruption of epithelial integrity and polarity, which can promote tissue disorganization and aberrant proliferation. Loss of apical–basal polarity and altered claudin expression may further facilitate tumor progression by enhancing exposure [26]. Claudins are the key determinants of epithelial permeability and can be broadly classified as either barrier-forming (“tight”) or pore-forming (“permeable”), depending on how they influence the permeability of the membrane [27]. Disruption of intercellular contacts leads to the loss of cell polarity, tissue disorganization, and increased exposure to various extracellular signals, including growth factors. Moreover, in the absence of apical–basal polarity, epithelial cells that receive growth signals beyond the apical domain tend to proliferate through out-of-plane divisions, a process driven by the misorientation of the mitotic axis [26].

The WGCNA reveals different modules, the turquoise module, composed of members of the let-7 and miR-200 families together with the miR-103/107 and miR-106a~363 clusters, appears to define a coordinated regulatory network involved in PCa progression. All miRNAs in this module were consistently up-regulated in TT and PTT relative to BT, suggesting an early and sustained role in tumor-associated reprogramming. let-7 family members are major players in the regulation of multiple genes involved in cell proliferation, differentiation, inflammation, and tumor progression and contribute greatly to the maintenance of the cellular homeostasis in normal prostatic cells [28]. The miR-200 family contributes to PCa pathogenesis by regulating ERG target genes, highlighting its role in TMPRSS2–ERG-driven tumor development [29], as well as in key processes such as epithelial–mesenchymal transition, cell adhesion, and invasion [30]. miR-103/107 and miR-106a~363 family members have been implicated in PCa through metabolic reprogramming, cell cycle progression, and TGF-β signaling, further supporting their role in shaping a permissive microenvironment for tumor initiation and progression [31,32].

Module preservation analysis further revealed that the turquoise and blue modules were moderately conserved between the tumor and peritumoral networks (Zsummary = 9.96 and 6.39, respectively), suggesting that while their miRNA composition is largely maintained, their internal connectivity patterns undergo moderate rewiring in the tumor context. In contrast, the yellow module, despite sharing 90% of its miRNAs with the tumor blue module, showed poor preservation (Zsummary = 0.64), indicating that this peritumoral module is not structurally conserved in the tumor microenvironment. The brown module was not preserved (Zsummary < 2), consistent with its identification as a PTT-specific module. These findings highlight that compositional overlap alone does not guarantee structural conservation, and that the peritumoral microenvironment harbors both shared and unique regulatory programs.

The functional enrichment analysis of predicted target genes revealed significant involvement in key oncogenic pathways, including PI3K–Akt, FoxO, p53, and HIF-1. These pathways are central to PCa biology, where they regulate tumor growth, survival, metabolic adaptation, and metastatic progression. Aberrant activation of the PI3K–Akt pathway by PTEN loss represents one of the most common molecular alterations in PCa and promotes proliferation, resistance to apoptosis, metabolic reprogramming, and therapy resistance [33,34]. Crosstalk between PI3K–Akt and downstream effectors such as FoxO transcription factors and HIF-1 further enhances tumor cell survival under stress conditions, including hypoxia [33,35,36,37].

In contrast, the blue and brown modules appear to reflect more nuanced regulatory layers within prostate cancer. miRNA-133a-3p and miRNA-326, both among the enriched miRNAS in blue module, have been described in PCa. miRNA-133a-3p is involved in cell proliferation, migration and invasion by targeting the epidermal growth factor receptor [38], and miRNA-326 is a tumor suppressor that plays crucial roles during various tumor development [39]. Meanwhile, the brown module was identified as a PTT-specific module that was not preserved in the tumor network. PCA of the brown module miRNAs revealed that PC2 separates TT samples (negative side) from PTT and BT samples (positive side), suggesting that this module captures a co-expression program that distinguishes the peritumoral/benign microenvironment from tumor tissue. The module contains a mixed composition of miRNAs with both oncogenic and tumor-suppressive functions, including miR-182-5p, miR-25-3p, miR-205-3p, miR-1-3p, and miR-338-3p. While miR-182-5p is significantly up-regulated in TT compared to BT (log_2_FC = 1.49, padj = 5.67 × 10^−19^) and TT compared to PTT (log_2_FC = 1.48, padj = 2.11 × 10^−18^), it is not differentially expressed between PTT and BT (log_2_FC = 0, padj = 1) (Table S1). Its presence in the brown module therefore reflects co-expression relationships specific to the peritumoral microenvironment rather than its absolute expression level. miR-182-5p and miR-25-3p have been shown to drive metastasis and cell survival by targeting the MITF suppressor and the PTEN/Akt pathway, respectively [40,41]. Conversely, the downregulation of tumor-suppressive miRNAs like miR-205-3p, miR-1-3p, and miR-338-3p is frequently associated with advanced disease stages and the induction of the EMT through the modulation of BCL2 and ZEB1/2 [42,43,44].

These findings illustrate that integrating miRNA expression profiling, co-expression network analysis, and functional enrichment provides a more comprehensive understanding of the regulatory mechanisms underlying molecular alterations in PTT. Integrative analytical strategies facilitate the biological interpretation of coordinated molecular networks and improve cancer stratification [45]. Recent studies combining spatial transcriptomics and single-nucleus RNA sequencing have demonstrated that the tumor microenvironment is organized into spatially distinct stromal and immune niches that actively regulate tumor progression and therapeutic response, further emphasizing the importance of characterizing the molecular landscape of the PTT compartment [46].

This study has several limitations that should be acknowledged. First, our findings were generated from a single discovery cohort and require validation in independent patient cohorts to confirm the reproducibility and generalizability of the identified miRNA signatures. Second, although functional enrichment and target prediction analyses provided biologically plausible insights, the predicted miRNA–mRNA interactions were not experimentally validated at the mRNA or protein level, and therefore should be interpreted as hypothesis-generating. Third, because bulk tissue was analyzed, some of the observed miRNA alterations may reflect changes in the cellular composition of the peritumoral microenvironment, including stromal and immune cells, rather than being exclusively derived from prostate epithelial cells. To minimize this possibility, PTT was strictly defined as morphologically benign glands located within 5 mm of malignant glands; whereas, BT was obtained from a separate paraffin block distant from the tumor. In addition, all tissue regions of interest were carefully reviewed and manually microdissected under light microscopy by an experienced cancer pathologist to ensure accurate histological classification of each tissue compartment.

## 4. Material and Methods

### 4.1. Patient Samples

The study population comprised 40 patients older than 18 years with histopathologically confirmed PCa, treated at the Colombian National Cancer Institute (INC) in Bogotá, Colombia, between 2011 and 2020. FFPE tissue blocks were retrieved from the surgical archives of the Pathology Laboratory following approval by the institutional ethics committee. For each case, hematoxylin–eosin (H&E)-stained slides were reviewed by an INC pathologist, who confirmed the diagnosis and assigned the corresponding Gleason grade group.

Patients were excluded if tissue samples were inadequate for molecular analysis, if they had received chemotherapy or radiotherapy prior to surgery, or if they had a personal history of a different malignant neoplasm.

For all 40 patients, at least one representative tissue sample was available, and paired specimens of benign tissue (BT), peritumoral tissue (PTT), and primary tumor tissue (TT) were selected whenever possible. PTT was defined as morphologically benign glands located within 5 mm of malignant glands; whereas, BT corresponded to benign glands selected from a separate paraffin block distant from the malignant tumor. Tissue regions of interest were carefully reviewed and manually dissected under light microscopy by an experienced cancer pathologist to ensure accurate histological classification of each tissue compartment. Clinicopathological variables were collected from pathology reports and medical records, including Gleason grade group, tumor location, and presence of extraprostatic extension, perineural invasion, and pathological TNM (pTNM) stage.

### 4.2. Ethics Statement

This study was reviewed and approved by the Research Ethics Committee of the National Institute of Cancerology (CEI-01032-22).

### 4.3. miRNA Isolation and Sequencing

BT, PTT, and TT sections were deparaffinized from FFPE tissue following a previously described protocol [47]. Total RNA was isolated using the AllPrep DNA/RNA Kit (QIAGEN, Venlo, The Netherlands) according to the manufacturer’s instructions. After RNA quantification and qualification, 100 ng were used to generate miRNA libraries using the QIAseq miRNA Library Kit (QIAGEN). Libraries were sequenced, and the resulting FASTQ files were processed using the QIAGEN GeneGlobe Data Analysis Center (https://geneglobe.qiagen.com/us/analyze, access date: 12 January 2026) for initial quality control, adapter trimming, alignment to miRBase v21, and generation of raw count matrices for each sample.

### 4.4. Data Preprocessing and Quality Control in R

The raw count matrix generated by GeneGlobe was imported into R (version 4.3.1) for analysis. A quality control (QC) pipeline was implemented to ensure data integrity following established guidelines for preprocessing next-generation sequencing data [48]. Low-expression miRNAs were filtered out by retaining only those with a count of at least 10 reads in a minimum of 10 samples, removing noise from downstream analyses. Sample-wise QC was performed using Principal Component Analysis (PCA) to identify potential outliers based on overall expression profiles. Library sizes and count distributions were visualized using boxplots and density plots to assess sequencing depth and batch effects before and after normalization. All QC steps and visualizations were conducted using the tidyverse (version 2.0.0) and DESeq2 (version 1.42.0) packages [49].

### 4.5. Differential Expression Analysis

Differential expression analysis was performed using the DESeq2 package (version 1.42.0) [49], which models count data using a negative binomial distribution and normalizes for library size and RNA composition through its median-of-ratios method.

To identify miRNAs associated with prostate carcinogenesis and the field effect, three pairwise comparisons were performed: (i) TT vs. BT; (ii) PTT vs. BT; (iii) TT vs. PTT.

To account for potential confounding factors and the matched nature of the samples, the DESeq2 model included patient ID as a blocking factor, along with Gleason grade group and age as covariates (design = ~Patient + Gleason_grade + Age + Tissue_Type). For each comparison, differentially expressed miRNAs (DEmiRs) were defined using an absolute log_2_ fold change (log_2_FC) threshold of ≥ 1 (equivalent to a 2-fold change) and a Benjamini–Hochberg (BH) adjusted *p*-value (False Discovery Rate, FDR) of <0.05.

### 4.6. Biological Interpretation and Functional Enrichment Analysis

To elucidate the biological implications of the observed miRNA dysregulation, we first separated differentially expressed miRNAs into up-regulated and down-regulated lists for each pairwise comparison (TT vs. BT; PTT vs. BT; and TT vs. PTT). For each list, experimentally validated and computationally predicted target genes were retrieved using miRWalk (version 3.0) [50], a comprehensive database that integrates miRNA-target interactions from multiple sources. To enhance the reliability of target predictions, we applied a stringent filtering criterion: only genes concurrently identified by all three integrated databases within miRWalk—namely, TargetScan, miRDB, and miRTarBase—were retained for subsequent analysis.

The resulting target gene lists for up-regulated and down-regulated miRNAs from each comparison were then independently subjected to functional enrichment analysis using the Database for Annotation, Visualization, and Integrated Discovery (DAVID) tool (version 2021) [51]. Overrepresentation analysis (ORA) was performed to identify significantly enriched Kyoto Encyclopedia of Genes and Genomes (KEGG) pathways. Enrichment significance was assessed using a modified Fisher’s exact test (EASE score), and *p*-values were adjusted for multiple testing using the Benjamini–Hochberg method. Pathways with an adjusted *p*-value < 0.05 were considered statistically significant and were retained for biological interpretation.

### 4.7. Weighted Gene Co-Expression Network Analysis (WGCNA)

To investigate the similarities and differences in miRNA co-expression architecture between the TT and PTT microenvironments relative to benign prostate tissue, we performed a weighted gene co-expression network analysis using the WGCNA R package (version 1.72-1) [52]. This analysis aimed to identify modules of co-expressed miRNAs that might reflect distinct biological processes operating in TT and PTT.

### 4.8. Selection of miRNAs for Network Construction

From the differential expression analyses described above (TT vs. BT; PTT vs. BT; and TT vs. PTT). We compiled a unified set of miRNAs that were significantly differentially expressed in at least one of the three pairwise comparisons. To capture a broader spectrum of potentially relevant transcriptional activity for network construction, we used a more permissive threshold of |log_2_FC| ≥ 0.584 (equivalent to a 1.5-fold change) and a Benjamini–Hochberg adjusted *p*-value (FDR) < 0.05. This 1.5-fold threshold was chosen because WGCNA can identify coordinated expression patterns (modules) that may include miRNAs with moderate but biologically relevant expression changes that would be missed by the more stringent 2-fold cutoff used for differential expression analysis.

### 4.9. Construction of Two Independent Networks

Two separate co-expression networks were constructed using the same set of selected miRNAs but with different sample subsets: (i) Tumor network: using all TT and BT samples. (ii) Peritumoral network: using all PTT and BT samples.

For each network, the workflow followed standard WGCNA guidelines. First, the biweight midcorrelation (bicor) was computed for all pairwise miRNA combinations, as this correlation measure is less sensitive to outliers. An appropriate soft-thresholding power (β) was selected by analyzing the scale-free topology fit index (R^2^) and mean connectivity as a function of different powers, choosing the lowest power that achieved an R^2^ > 0.8. The adjacency matrix was then calculated by raising the bicor matrix to the selected soft power. Subsequently, the topological overlap measure (TOM) was derived from the adjacency matrix to quantify the relative interconnectedness of miRNAs, and the corresponding dissimilarity (1-TOM) was used as input for hierarchical clustering.

Module detection was performed using dynamic tree cutting on the hierarchical dendrogram, with a minimum module size of 5 miRNAs. To obtain relatively large and distinct modules, we merged modules whose eigengenes were highly correlated (Pearson correlation > 0.75). For each resulting module, we computed the module eigengene (ME) as the first principal component of the miRNA expression profiles within the module, which summarizes the overall expression pattern of that module.

### 4.10. Module–Trait Relationships

To associate modules with the tissue types (TT or PTT vs. BT), we correlated the module eigengenes with a binary trait variable (e.g., tumor = 1, benign = 0) using Pearson correlation. Modules showing significant correlations (*p* < 0.05) were considered candidate modules potentially linked to the pathological state.

### 4.11. Comparison of Network Architectures

To assess the conservation of co-expression patterns between the TT and PTT networks, we performed a module preservation analysis using the WGCNA function module Preservation. This approach calculates composite preservation statistics (Zsummary and medianRank) by permuting sample labels (100 permutations). Modules with Zsummary > 10 were considered strongly preserved, those with 2 < Zsummary < 10 were weakly preserved, and those with Zsummary < 2 as not preserved. This analysis allowed us to identify modules that are conserved (i.e., present in both networks) and modules that are specific to one condition.

Additionally, we compared the module composition directly by examining the overlap of miRNAs between modules of the two networks. For each module in the tumor network, we identified the corresponding module in the adjacent network that shared the highest number of miRNAs (hypergeometric test, adjusted *p* < 0.05). Modules that did not show significant overlap with any module in the other network were considered condition-specific. Finally, we examined the biological relevance of condition-specific and conserved modules by subjecting the miRNA members to pathway enrichment analysis using the same miRWalk/DAVID pipeline described above.

### 4.12. Principal Component Analysis of Module-Specific miRNAs and Subsequent Enrichment

To further dissect the transcriptional architecture distinguishing TT, PTT, and BTs, we leveraged the WGCNA-derived module assignments to guide a focused Principal Component Analysis (PCA). Specifically, we separated miRNAs into two distinct sets based on their module preservation status: (i) miRNAs belonging to conserved modules (i.e., those preserved between TT and PTT networks, Zsummary > 2) and (ii) miRNAs belonging to non-conserved or condition-specific modules (i.e., those with Zsummary < 2 or showing significant module–trait relationships unique to one network). For each of these two miRNA sets, we performed PCA on the normalized expression values (variance-stabilizing transformation from DESeq2) across all samples (TT, PTT, and BT), using the prcomp function in R with centering and scaling.

The resulting two-dimensional projections were visually inspected to assess the capacity of each miRNA set to discriminate the three tissue types. Particular attention was paid to projections that revealed either (a) clear separation between TT and PTT samples, suggesting that the corresponding miRNA set captures transcriptional differences between these two microenvironments, or (b) clustering of TT and PTT samples together but separated from BT samples, indicating shared alterations relative to BT.

For those PCA projections exhibiting biologically meaningful separation, we extracted the loading vectors (i.e., the contributions of individual miRNAs to the principal components). To identify the most influential drivers of the observed separation, we selected the top 10% of miRNAs with the highest absolute loading values along the principal components of interest (typically PC1 and PC2, which collectively explained the largest proportion of variance). This threshold ensured that only the most discriminative miRNAs were retained for downstream functional analysis.

Given the reduced number of miRNAs in these selected sets, we opted for a focused target prediction approach using the TargetScan database integrated within miRWalk (version 3.0) [50]. TargetScan was chosen due to its high-confidence predictions based on seed region complementarity and evolutionary conservation, which is particularly suitable when working with small, curated miRNA lists. Predicted target genes were then subjected to overrepresentation analysis (ORA) using the enrichment analysis module available in miRWalk [50] to identify overrepresented KEGG pathways. Enrichment significance was assessed using a modified Fisher’s exact test with Benjamini–Hochberg adjustment for multiple testing, retaining pathways with an adjusted *p*-value < 0.05 for biological interpretation. This approach allowed us to link condition-specific co-expression modules and their most influential miRNAs to distinct biological pathways potentially driving the molecular continuum from benign to tumor through adjacent tissue.

## 5. Conclusions

Our findings demonstrate that PTT should not be routinely considered equivalent to benign tissue in molecular studies for prostate cancer. PTT exhibits a tumor-like molecular profile despite its benign histological appearance, supporting the concept of field cancerization. This suggests that PTT is not just a “soft tumor” but has active stress response mechanisms or epigenetic remodeling that the tumor has already lost or outgrown. The substantial overlap in miRNA expression between PTT and TT, together with the identification of shared oncogenic pathways, highlights the presence of early molecular alterations that may contribute to tumor initiation, progression, and recurrence. Notably, the enrichment of pathways related to epigenetic regulation, metabolic reprogramming, and cellular stress responses underscores the complexity of tumor-associated reprogramming beyond the tumor boundary. Furthermore, the coordinated miRNA networks identified, reinforce their central role in regulating key processes such as proliferation, EMT, and microenvironmental interactions.

## Figures and Tables

**Table 1:** Clinicopathological characteristics of patients with PCa stratified by Gleason grade group.

Characteristic	Global (n = 37)	G1 (n = 6)	G2 (n = 8)	G3 (n = 7)	G4 (n = 8)	G5 (n = 8)
Age at diagnosis (years)						
Median (range)	65.4 (48.2–79.1)	63.8 (55.9–79.1)	68.8 (60.5–75.7)	61.2 (53.0–71.8)	64.1 (54.4–78.6)	67.0 (48.2–74.9)
PSA at diagnosis (ng/mL)						
Median (range)	10.3 (2.4–43.0)	5.4 (3.9–17.5)	8.4 (5.4–24.0)	12.5 (7.2–43.0)	9.6 (2.4–25.0)	14.6 (6.9–42.8)
Perineural invasion						
Present	22 (59.5%)	3 (50.0%)	6 (75.0%)	2 (28.6%)	4 (50.0%)	7 (87.5%)
Absent	15 (40.5%)	3 (50.0%)	2 (25.0%)	5 (71.4%)	4 (50.0%)	1 (12.5%)
Extraprostatic extension						
Present	23 (62.2%)	4 (66.7%)	2 (25.0%)	3 (42.9%)	8 (100%)	6 (75.0%)
Absent	14 (37.8%)	2 (33.3%)	6 (75.0%)	4 (57.1%)	0 (0%)	2 (25.0%)
Vital status						
Alive	28 (75.7%)	3 (50.0%)	5 (62.5%)	7 (100%)	7 (87.5%)	6 (75.0%)
Deceased	5 (13.5%)	0 (0%)	2 (25.0%)	0 (0%)	1 (12.5%)	2 (25.0%)
Not available	4 (10.8%)	3 (50.0%)	1 (12.5%)	0 (0%)	0 (0%)	0 (0%)

Abbreviations: G, Gleason grade; PSA, prostate-specific antigen.

**Table 2:** KEGG pathways uniquely enriched in PTT from up-regulated miRNAs.

Pathways	Count	Fold Enrichment	FDR
Longevity regulating pathway—multiple species	12	2.79	0.0212
Tight junction	23	1.95	0.0212
Polycomb Repressive Complex	14	2.44	0.0241
Mitophagy–animal	16	2.2	0.0295
Protein processing in the endoplasmic reticulum	22	1.87	0.0342
Chemical carcinogenesis—receptor activation	26	1.75	0.0357
Transcriptional misregulation in cancer	24	1.75	0.0445

**Table 3:** Module overlap between TT and PTT co-expression networks.

PTT_Module	TT_Module	PTT_Size	TT_Size	Overlap_Count	Percent_Overlap	Odds_Ratio	*p*_Value	FDR	Z Summary
blue	blue	56	99	49	87.5	16.565	8.67 × 10^−15^	3.03 × 10^−14^	6.39
turquoise	turquoise	66	73	52	78.79	24.036	2.44 × 10^−21^	1.71 × 10^−20^	9.96
yellow	blue	40	99	36	90	17.361	3.04 × 10^−11^	7.09 × 10^−11^	0.64
brown	turquoise	50	73	16	32	0.983	5.84 × 10^−1^	1	--
brown	blue	50	99	3	6	0.054	1.00 × 10^0^	1	--
turquoise	blue	66	99	5	7.58	0.058	1.00 × 10^0^	1	--
yellow	turquoise	40	73	1	2.5	0.041	1.00 × 10^0^	1	--

## Data Availability

The original contributions presented in this study are included in the article/Supplementary Materials. Further inquiries can be directed to the corresponding author.

## Supplementary Materials

The following supporting information can be downloaded at: https://www.mdpi.com/article/10.3390/ijms27156637/s1.
